# Adequate Platelet Function Inhibition Confirmed by Two Inductive Agents Predicts Lower Recurrence of Ischemic Stroke/Transient Ischemic Attack

**DOI:** 10.1155/2017/3504950

**Published:** 2017-08-24

**Authors:** Lulu Zhang, Xiaowei Hu, Juehua Zhu, Xiuying Cai, Yan Kong, Hui Wang, Shanshan Diao, Hongru Zhao, Jianhua Jiang, Dapeng Wang, Quanquan Zhang, Yiren Qin, Wei Yue, Qi Fang

**Affiliations:** ^1^Department of Neurology, The First Affiliated Hospital of Soochow University, No. 899, Pinghai Road, Suzhou, Jiangsu 215006, China; ^2^Department of Neurology, Tianjin Huanhu Hospital, No. 122, Qixiangtai Road, Hexi District, Tianjin 300060, China

## Abstract

**Background:**

The correlation between platelet function and recurrent ischemic stroke or TIA remains uncertain.

**Objective:**

To investigate two inductive agents to detect platelet functions and assess associations with recurrent ischemic stroke/TIA.

**Method:**

The study included 738 ischemic stroke/TIA patients. On days 0, 3, and 9 after antiplatelet therapy, platelet function tests were determined by maximum aggregation rate (MAR) using a PL-11 platelet function analyzer and phase matching reagents. Two induction agents were used: arachidonic acid (AA) and adenosine diphosphate (ADP). At 3-month follow-up, recurrence of stroke/TIA was recorded.

**Result:**

Cut-off values of adequate platelet function inhibition were MAR_ADP_ < 35% and MAR_AA_ < 35%. Data showed that antiplatelet therapy could reduce the maximum aggregation rate. More importantly, adequate platelet function inhibition of either MAR_ADP_ or MAR_AA_ was not associated with the recurrence of stroke/TIA, but adequate platelet function inhibition of not only MAR_ADP_ but also MAR_AA_ predicts lower recurrence (0/121 (0.00%) versus 18/459 (3.92%),* P* = 0.0188).

**Conclusion:**

The platelet function tested by PL-11 demonstrated that adequate inhibition of both MAR_ADP_ and MAR_AA_ could predict lower risk of ischemic stroke/TIA recurrence.

## 1. Introduction

Stroke is the second most common cause of death and is a major cause of disability worldwide [1, 2]. The prognosis of recurrent stroke is worse than first-ever stroke [3, 4]. Antiplatelet therapy is the cornerstone of the secondary prevention of ischemic stroke and transient ischemic attack (TIA) [5]. However, a large number of stroke/TIA patients still experience another cerebral vascular event despite sustained antiplatelet therapy. On average, the annual risk for future ischemic stroke after an initial ischemic stroke/TIA is 3% to 4% [6]. Some researchers interpret such individual difference to antiplatelet drugs as “aspirin/clopidogrel resistance” [7–9]. Therefore, appropriate platelet function measurement to predict aspirin/clopidogrel efficacy is necessary to guide the precise stroke treatment. The current study is a multicenter study designed to evaluate the platelets' function before and after antiplatelet therapy and analyze the relationship between qualified platelet inhibition and stroke recurrence in the Chinese population.

## 2. Methods

### 2.1. Study Design and Patients

The current study is a prospective trial that enrolled 738 patients from 13 stroke centers between October 2014 and December 2015 in China. Inclusion criteria were as follows: (1) age: ≥18 years old and ≤80 years old; (2) transient ischemic attack (TIA) for the first time, which was defined as a transient episode of neurological dysfunction caused by focal brain or retinal ischemia, without acute infarction symptoms; (3) ischemic stroke for the first time or recurrent patients with modified Rankin Scale ≤2 (ischemic stroke was defined as a sudden neurological deficit, and the infarct site was assessed by magnetic resonance imaging); (4) need for antiplatelet therapy: 100 mg aspirin or 75 mg clopidogrel as monoantiplatelet therapy is initiated within 24 hours after the onset; patients with the following were excluded from the study: (1) intracranial venous thrombosis, (2) cerebral hemorrhage, (3) cerebral embolism, (4) brain tumors, and (5) end-stage severe disease. The main endpoint during the 3-month follow-up was the recurrence of stroke/TIA, bleeding events including cerebral hemorrhage and gastrointestinal bleeding, and other bleeding events.

### 2.2. Participant Stroke Centers

1. The First Affiliated Hospital of Soochow University
2. Tianjin Huanhu Hospital
3. Peking Union Medical College Hospital (West)
4. Weihai Municipal Hospital
5. Changhai Hospital
6. Shanghai East Hospital
7. Lanzhou University Second Hospital
8. Zhuhai People's Hospital
9. Harrison International Peace Hospital
10. The Second Hospital of Tianjin Medical University
11. Shijiazhuang Third Hospital
12. Affiliated Hospital of North Sichuan Medical College
13. Shandong Provincial Hospital

### 2.3. Key Technology

PL-11 platelet function analyzer (SINNOWA Medical Science & Technology Co., Nanjing, China) is a new point-of-care apparatus for platelet function analysis via an automated impedance technique. Correlations among PL-11 and another three major assays (light transmission aggregometry (LTA), VerifyNow aspirin system, and thromboelastography (TEG)) suggested the ability of PL-11 to assess platelet function.

### 2.4. Treatment Protocols

All enrolled patients were given aspirin 100 mg/d or clopidogrel 75 mg/d. The medicine would not be changed during the experiment unless patients encountered hemorrhagic or ischemic events or withdrew from the experiment. At the same time, other antiplatelet medicines could not be provided, including Chinese patent medicine containing ingredients like Folium Ginkgo, Salvia Miltiorrhiza, Pseudoginseng, and so on.

### 2.5. Standard Protocol Approvals, Registration, and Patient Consent

The study protocol was approved by the ethics committee at each study center. Written informed consent was obtained from all participants or their proxies. This trial has been registered in the Chinese Clinical Trials Registry and the registration number is ChiCTR-OCH-14005238.

### 2.6. Sample Collection and Processing

Antecubital vein blood samples were collected with 3.8% sodium citrate in tubes for monitoring platelet function in all subjects on the day patients were admitted before antiplatelet treatment and 3 days and 9 days after antiplatelet therapy. Blood samples should be stored at room temperature before being tested. The whole procedure required being performed within 2 hours after sampling. Platelet aggregation was detected using PL-11 platelet function analyzer [10] (SINNOWA Medical Science & Technology Co., Nanjing, China).

The whole procedure was automatically done after transferring 500 ml of citrated blood sample into a polycarbonate tube and inserting it into the detecting position. The blood sample in the polycarbonate tube was mixed gently during the whole testing process. Platelet count was detected in duplicate at the start and the mean value of platelet count was set as the baseline. There was a short interval between each test point for system cleaning. 40 *μ*l of adenosine diphosphate (ADP, 50 *μ*mol/L) and arachidonic acid (AA, 2 mg/ml) were separately trickled into the blood sample after the second detecting time. The single platelet counting dropped when aggregates formed became too large to be counted as single platelets. PL-11 counted platelets several times until it detected the lowest level. The whole process was finished within 15 min (six detecting times). The system calculated the maximal platelet aggregation ratio according to the following formula:

(1) $$\begin{array}{c} MAR\%=\frac{\left(1st\;\;platelet\;\;count+2nd\;\;platelet\;\;count\right)/2-lowest\;\;platelet\;\;count}{\left(1st\;\;platelet\;\;count+2nd\;\;platelet\;\;count\right)/2}. \end{array}$$

The corresponding maximum aggregation rate of the platelet by each inductive agent was recorded as MAR_AA_ and MAR_ADP_.

### 2.7. Statistical Analysis

Baseline characteristics were compared between the ending group (recurrence of ischemic stroke/TIA) and no ending group (nonrecurrence of ischemic stroke/TIA). Continuous variables are presented as mean (standard deviation) and differences were compared using the analysis of Wilcoxon test. Categorical variables are presented as counts (proportions). Differences were compared using the Fisher test. All tests were 2-sided at a significance level of *P* ≤ 0.05 and were performed using SAS software, Version 9.4.

## 3. Results

### 3.1. Characteristics of the Patients

From October 2014 through December 2015, we enrolled 738 patients.

Baseline characteristics of patients by recurrent ischemic stroke/TIA at 3-month follow-up were well matched (Table 1).

### 3.2. Antiplatelet Therapy Reduced MAR

**Figure 1:**
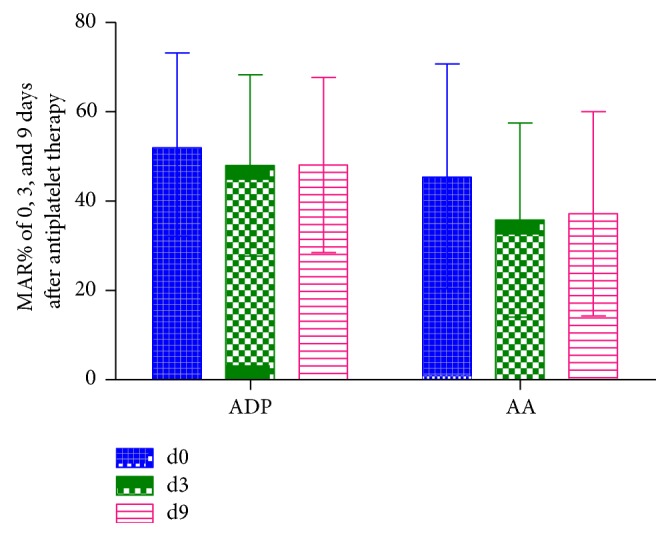
MAR% of 0, 3, and 9 days after antiplatelet therapy induced by ADP and AA.

**Figure 2:**
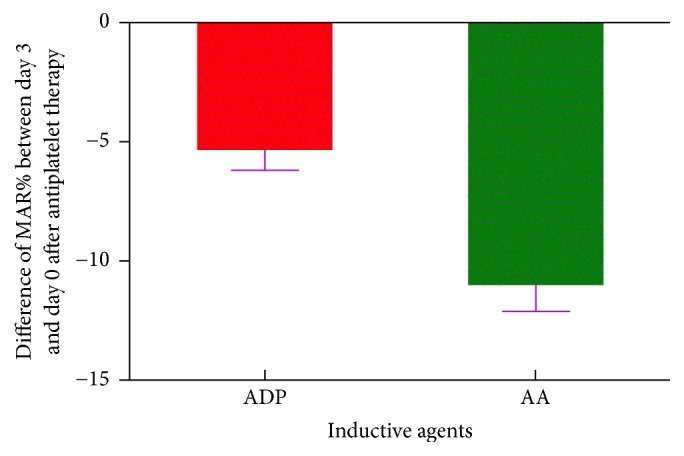
Difference of MAR% between day 3 and day 0 after antiplatelet therapy induced by ADP and AA.

**Figure 3:**
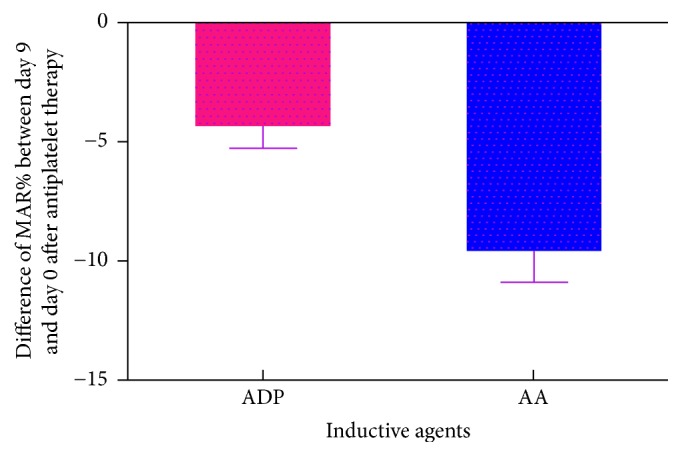
Difference of MAR% between day 9 and day 0 after antiplatelet therapy induced by ADP and AA.

Compared with baseline (Figure 1), MAR_ADP_ was decreased by 9.71% on day 3 (Figure 2, *n* = 672, 47.99% ± 20.30% versus 53.15% ± 20.72%, *P* < 0.0001) and by 9.48% on day 9 (Figure 3, *N* = 581, 48.11% ± 19.62% versus 53.15% ± 20.72%, *P* < 0.0001) after antiplatelet therapy. MAR_AA_ was decreased by 22.37% on day 3 (Figure 2, *n* = 664, 35.77% ± 21.72 versus 46.08% ± 25.29%, *P* < 0.0001) and by 19.34% on day 9 (Figure 3, *N* = 572, 37.17% ± 22.84% versus 46.08% ± 25.29%, *P* < 0.0001) after antiplatelet therapy.

### 3.3. Association between Adequate Platelet Function Inhibition and Recurrence of Stroke/TIA at 3-Month Follow-Up

The cut-off values of adequate platelet function inhibition were MAR_ADP_ < 35% and MAR_AA_ < 35%. Based on these criteria, we divided patients into adequate platelet function inhibition and inadequate inhibition groups. Recurrence of ischemic stroke/TIA at 3-month follow-up was compared between the two groups. When being grouped based on MAR_ADP_, for patients with MAR_ADP_ < 35%, the recurrence cases were 2 at 3-month follow-up (Table 2, 2/172 (1.16%)), while for patients with MAR_ADP_ ≥ 35%, the recurrence cases were 16 at 3-month follow-up (Table 2, 16/417 (3.84%)). Although the recurrent cases of the adequate platelet function inhibition group were fewer, there was no significant difference between the two groups (*P* = 0.0864). When being grouped based on MAR_AA_, the recurrence rate was not significantly different either (Table 2, MAR_AA_ < 35% versus MAR_AA_ ≥ 35%, 2.90% (10/345) versus 3.31% (8/242), *P* = 0.7782).

When setting the stricter criteria, take not only MAR_ADP_ < 35% but also MAR_AA_ < 35% as adequate platelet function inhibition. Based on these stricter criteria, we divided patients into adequate platelet function inhibition and inadequate inhibition groups and compared recurrence of ischemic stroke/TIA between the two groups. At 3-month follow-up, 0.00% (0/121) of the patients experienced recurrence of stroke/TIA in the group of adequate platelet function inhibition, while for the inadequate inhibition group, 18/459 (3.92%) experienced recurrent stroke/TIA. The recurrence rate was significantly different between the two groups (Table 2, *P* = 0.0188).

## 4. Discussion

The role of antiplatelet therapy in stroke prevention was well documented, especially for secondary prevention [11–13]. But it was also confirmed that there were still a considerable number of patients with ischemic stroke/TIA recurrence even if on treatment of single antiplatelet therapy with aspirin or clopidogrel [5]. This phenomenon may be associated with platelet function. Our study showed that antiplatelet therapy could reduce both MAR_ADP_ and MAR_AA_ in patients of ischemic stroke/TIA. At 3-month follow-up, neither adequate inhibition of MAR_ADP_ nor MAR_AA_ was associated with the recurrence of ischemic stroke/TIA, but adequate inhibition of not only MAR_ADP_ but also MAR_AA_ could predict lower recurrence of ischemic stroke/TIA.

Our study found that MAR_AA_ and MAR_ADP_ significantly decreased in patients of ischemic stroke/TIA after antiplatelet therapy which was consistent with a large number of studies and clinical observations since 2002.

A number of studies had concentrated on the association between platelet function and ischemic events [14]. ARMYDA-Pro [15] and including popular research [16] which was the largest assessment of the predictive value of platelet function tests so far all showed that platelet function was significantly correlated with ischemic vascular outcome. However, several large-scale studies have denied the correlation. TRILOGY-ACS subgroup [17, 18] analysis showed that prasugrel could significantly reduce platelet aggregation but could not reduce cardiovascular mortality, nonfatal myocardial infarction, or stroke within 30 months. Translate-POPs [19] research randomly assigned ACS patients to a strategy of platelet function monitoring, with drug adjustment in patients who had poor responses to antiplatelet therapy, or to a conventional strategy without monitoring and drug adjustment. This study showed no significant improvements in clinical outcomes between the two groups. Similar negative results also had been confirmed by ARCTIC research [20]. Several reasons may account for the different results: (1) platelet function was detected by VerifyNow P2Y12 in some researches, but the method was proven to have low sensitivity [20, 21]; (2) platelets had 6-7 kinds of different receptors and all above researches only measured platelet function by ADP inductive agent, so this may not bring us accurate information [22]. Our study used MAR_AA_ and MAR_ADP_ to evaluate platelet function by PL-11 platelet function analyzer, demonstrating that the single adequate inhibition of either MAR_ADP_ or MAR_AA_ was not associated with the decreased risk of recurrent ischemic stroke/TIA; however, adequate inhibition of both MAR_ADP_ and MAR_AA_ could predict the lower risk of ischemic stroke/TIA recurrence.

The shortcomings of this study are as follows: (1) There is a limitation of MARAA and MARADP by PL-11: the cut-off values of adequate platelet function inhibition by different inductive agents were not confirmed, requiring large cohort studies; (2) the study did not involve adjusting the antiplatelet therapy for ineffective inhibition of platelet function.

## Figures and Tables

**Table 1:** Baseline characteristics of patients by recurrent ischemic stroke/TIA at a 3-month follow-up.

	Ending	No ending	*P* value
Sex (female), number (%)	14 (66.67)	492 (68.62)	0.8141
Age, years, mean (SD)	63.71 ± 9.01	62.54 ± 10.62	0.3503
Smoking habit, number (%)	7 (33.33)	273 (38.08)	0.8204
SBP, mmHg, mean (SD)	143.76 ± 19.91	147.18 ± 21.89	0.3506
DBP, mmHg, mean (SD)	81.67 ± 9.69	85.35 ± 12.75	0.2093
FPG, mmol/l, mean (SD)	7.21 ± 2.78	7.49 ± 12.97	0.1449
TC, mmol/l, mean (SD)	4.80 ± 1.20	5.99 ± 14.29	0.7442
TG, mmol/l, mean (SD)	1.59 ± 0.54	1.58 ± 1.34	0.1750
BMI, kg/m^2^, mean (SD)	23.52 ± 2.03	24.34 ± 2.85	0.1124
Cr, mmol/l, mean (SD)	72.39 ± 29.61	72.94 ± 37.33	0.8156
NIHSS points, mean (SD)^**∗**^	6.65 ± 4.82	5.25 ± 3.74	0.2866
ABCD points, mean (SD)^#^	3.25 ± 1.71	3.15 ± 1.34	0.8077

^*∗*^Compare NIHSS points for patients of ischemic stroke. ^#^Compare ABCD points for patients of transient ischemic attack.

**Table 2:** Association of inhibited platelet aggregation with the recurrent ischemic stroke/TIA within a 3-month follow-up.

Inductive agent	Groups (MAR)	Ending (%)	No ending (%)	Numbers	*P* value
ADP	≥35%	16 (3.84)	401 (96.16)	417	0.0864
	<35%	2 (1.16)	170 (98.84)	172	
AA	≥35%	8 (3.31)	234 (96.69)	242	0.7782
	<35%	10 (2.90)	335 (97.10)	345	
ADP + AA	ADP ≥ 35% or AA ≥ 35%	18 (3.92)	441 (96.08)	459	0.0188
	ADP < 35% and AA < 35%	0 (0.00)	121 (100.00)	121	
